# The significance of feeling safe for resilience of adolescents in sub-Saharan Africa

**DOI:** 10.3389/fpsyg.2023.1183748

**Published:** 2023-08-17

**Authors:** Monica Bandeira, Marien A. Graham, Liesel Ebersöhn

**Affiliations:** ^1^Centre for the Study of Resilience and Department of Educational Psychology University of Pretoria, Pretoria, South Africa; ^2^Department of Science, Mathematics and Technology Education, University of Pretoria, Pretoria, South Africa

**Keywords:** adolescence, resilience, mental health, sub-Saharan Africa, depression, self-esteem, safety

## Abstract

**Introduction:**

Adolescents in sub-Saharan Africa (SSA) are exposed to several challenges and risk factors, linked to historical legacies. Sub-Saharan Africa has one of the highest rates of poverty and inequality in the world, is one of the regions most negatively affected by climate change, performs poorly on many health measures, and has high rates of different forms of violence, especially gender-based violence. These contextual challenges impact adolescent mental health outcomes, preventing them to access resilience-enabling pathways that support positive outcomes despite adversity. This study aimed to contribute to knowledge generation on resilience of young people in the understudied SSA region by investigating which variables directly (or indirectly) affect the resilience of adolescents.

**Methods:**

Purposive sampling was used to collect quantitative survey data from 3,312 adolescents (females = 1,818; males = 1,494) between the ages of 12 and 20 years, participating in interventions implemented by a non-governmental organization, the Regional Psychosocial Support Initiative. Data were collected in Angola (385, 11.6%), Eswatini (128, 3.9%), Kenya (390, 11.8%), Lesotho (349, 10.5%), Mozambique (478, 14.4%), Namibia (296, 8.9%), South Africa (771, 23.3%), Uganda (201, 6.1%), and Zambia (314, 9.5%). The survey collected data on socio-demographic status, resilience (CYRM-R), depression (PHQ-9), self-esteem (Rosenberg Self-Esteem Scale) and feelings of safety (self-developed scale). Mental health was defined as lower levels of depression, higher levels of self-esteem and higher levels of feeling safe. A mediation analysis was conducted to investigate the relationship between the predictors (the socio-demographic variables) and the output (resilience), with the mediators being depression, self-esteem and feeling safe (which all link to mental health).

**Results:**

This study contributes to a gap in knowledge on country-level comparative evidence on significant predictors that impact resilience outcomes (directly or indirectly) for adolescents in sub-Saharan African countries. The results indicate that, when considering all countries collectively, feeling safe is the only predictor that has a significant direct effect on overall resilience and personal resilience, but not on caregiver resilience. When considering each country separately, feeling safe has a direct effect on overall, personal and caregiver resilience for all countries; but not for South Africa and Mozambique.

**Discussion:**

The results provide evidence on which to craft youth development interventions by measuring mediators (depression, self-esteem and feeling safe) and resilience for adolescents in sub-Saharan Africa. The overall results of the present paper point toward a contextually relevant pathway to supporting their resilience, namely, the need to systemically target the creation and/or strengthening of structures that enable adolescents to feel safe.

## 1. Introduction

Of 1.3 billion adolescents world-wide in the United Nations Children’s Fund [UNICEF] (2022), an estimated nine out of ten live in the developing world (Gupta et al., 2014), with almost one quarter (23%) of the population (aged between 10 and 19 years of age)–the greatest proportion of the population (United Nations Children’s Fund [UNICEF], 2019a)–in sub-Saharan Africa (SSA). Despite this, the available literature on youth development in sub-Saharan Africa is limited.

During adolescence, individuals acquire physical, cognitive, emotional, and social resources that become the foundation for their future health and wellbeing (Patton et al., 2016). However, this period of turmoil is characterized by adolescents being confronted by many changes, which increase their vulnerability to risks and may inhibit their capacity to manage challenges (Ogden and Hagen, 2018). Mental illness has become a leading cause of death and disability for adolescents (Vigo et al., 2016; Uddin et al., 2019), with many studies associating mental health outcomes to resilience (Konaszewski et al., 2021; Mesman et al., 2021).

Whereas there has been an increase in the global incidence of mental disorders, and more so in low-and-middle-income countries (Patel et al., 2018), the problem is particularly amplified during adolescence. The World Health Organization (WHO) indicates that up to one in five adolescents will experience a mental disorder each year, that self-harm is the third leading cause of death for adolescents, and that depression is among the leading causes of disability (World Health Organization [WHO], 2014).

While various studies on mental health exist, Glozah (2015) found that most of the knowledge about the psychosocial context of adolescent health and wellbeing is based on western samples. Existing research tends to report on data from individual countries rather than across countries or across countries in the global north or outside of sub-Saharan Africa (Gloster et al., 2020; Campbell et al., 2021; Jefferies et al., 2021a,b; White et al., 2021) or between countries in the global north and sub-Saharan Africa (Alonso et al., 2018; Reed et al., 2018; Wu et al., 2018; Yu, 2018; Alzueta et al., 2021; Höltge et al., 2021) and not exclusively in sub-Saharan Africa, highlighting the severe lack of research on mental health in the region (Sankoh et al., 2018).

A systematic review exploring mental health problems of adolescents in sub-Saharan Africa highlights the enormity of the problem, with prevalence rates of 26.9% for depression, 29.8% for anxiety, 40.8% for emotional or behavioral problems, 21.5% for post-traumatic stress disorder (PTSD), and 20.8% for suicidal ideation (Jörns-Presentati et al., 2021). Young people living with HIV in sub-Saharan Africa are particularly at risk of common mental health disorders, especially depression and anxiety (Too et al., 2021), with 25% of HIV-positive adolescents having a psychiatric disorder and up to 50% showing emotional or behavioral difficulties or psychological distress (Dessauvagie et al., 2020).

Studies that do examine comparative mental health or resilience in sub-Saharan Africa tend to be qualitative (Lund, 2010; Bird et al., 2011; Esan et al., 2019) and include few countries (Esan et al., 2019; Kuo et al., 2019), or are based on systematic reviews (Pedersen et al., 2019; Theron, 2020a; Chen et al., 2021; Scharpf et al., 2021; Trudell et al., 2021). Existing literature also tends to focus either exclusively on mental health (Auerbach et al., 2018; Sankoh et al., 2018; Yu, 2018; Żemojtel-Piotrowska et al., 2018; Chen et al., 2021) or resilience (Ungar and Liebenberg, 2011; Theron, 2020b; Höltge et al., 2021; Jefferies et al., 2021b).

The high incidence of challenged adolescent mental health requires early intervention (Catalano et al., 2012; Collishaw, 2015; Das et al., 2016). Yet, limited attention has been paid to factors that significantly affect adolescent mental health and wellbeing (United Nations Children’s Fund [UNICEF], 2019b). Rather, existing studies foreground sexual reproductive health, with scant insight on the numerous factors that negatively impact the health and wellbeing of adolescents (Hervish and Clifton, 2012; Kabiru et al., 2013).

The high demand on intervention to support positive adolescent mental health points to an ever-widening mental health treatment gap for this group in sub-Saharan Africa (Owen et al., 2016). The Lancet Commission on global mental health and sustainable development suggests that a shift in the focus of the global mental health agenda is required “reducing the contribution of mental disorders to the improvement of mental health for whole populations and reducing the contribution of mental disorders to the global burden of disease” (Patel et al., 2018, p. 1). Prevention initiatives during this stage may help decrease problem severity, deter comorbidity, and reduce the chances of new problems emerging (Ogden and Hagen, 2018).

Despite adolescents comprising approximately one-quarter of the population of sub-Saharan Africa, the health of adolescents has been understudied in this region (Ross, 2021). Although some knowledge exists, gaps remain in the exploration of resilience-enabling pathways which enhance mental health, as well as how these may be similar or different across low-and-middle-income countries in sub-Saharan Africa (Bosqui and Marshoud, 2018). The purpose of the article is to use comparative evidence (across sub-Saharan countries, gender and age) of adolescent resilience and mental health outcomes to craft an evidence-based resilience framework to direct relevant support for youth development in sub-Saharan Africa. As such, the question directing this article is: Which predictors have a direct effect on mental health (as mediator) or direct or indirect effect on resilience (as outcome) in sub-Saharan Africa? The mediator (mental health) was measured by depression, self-esteem and safety, as many studies have shown that mental health is affected by depression (McLeod et al., 2016; Aluh et al., 2018), self-esteem (Keane and Loades, 2017; Minev et al., 2018) and safety (Mori et al., 2021; Fossum et al., 2023).

In the current study, a mediation analysis was conducted to investigate the relationship between the predictors (the socio-demographic variables) and the output (resilience), with the mediators being depression, self-esteem and feeling safe (which all link to mental health). Most commonly, resilience would be operationalized within a statistical model as a moderator or a mediator - that is, interrupting the expected association between some adversity and poorer outcome in the presence of higher levels of resilience. For resilience as mediator/moderator, Karatzias et al. (2017) assessed resilience and depression as mediators between traumatic life events (predictors) and subjective physical and mental health (outcomes) and found that resilience mediated the relationships between traumatic life events and subjective physical and mental health. More recently, Lin et al. (2020) assessed resilience as mediator between bullying experiences (both victimization and perpetration; predictors) and mental health (outcome) in China and Germany and found that personal resilience partially mediated the influence of victimization on mental health in both countries. However, some literature has considered resilience as outcome variable, for example, Lancaster and Callaghan (2022) assessed exercise, location, life-orientation, mental health, and sleep quality as key moderators and mediators of resilience.

In accordance with recent studies such as the one by Lancaster and Callaghan (2022), we were interested in studying the mediating role of mental health on resilience, as this is a topic that has not been researched much. We considered depression (Sit et al., 2022), self-esteem (Preston and Rew, 2022) and feeling safe (Luu et al., 2021) as mediators as these have been shown to be significantly related to mental health and tools and systems to improve mental health have been understudied in low-resource environments such as SSA (Goodman et al., 2021).

## 2. Literature review

### 2.1. Contextual challenges impacting on adolescent development in sub-Saharan Africa

Adolescence is a critical stage of psychosocial development during which individuals are vulnerable to numerous risks while at the same time being exposed to opportunities for development. Adolescents in sub-Saharan Africa are particularly vulnerable given the contextual challenges they face, evidenced by the knowledge available on depression on the continent (Sherr and Cluver, 2017; Kulisewa et al., 2019). Global South-based evidence is needed given the context challenges in which many adolescents in sub-Saharan Africa are living. Many of these challenges are rooted within the historical legacies of deeply held pre-colonial ethnic-specific political centralisation; the damaging period of slave trade; the artificial creation of colonial borders by Europeans; and the extractive and oppressive period of colonization (Michalopoulos and Papaioannou, 2020). Adolescents in sub-Saharan Africa are growing up in complex environments largely shaped by history. From a chronosystems perspective, individuals are shaped by the historical times and events they experience over their lifetime; when in their life course transitions and events happen; the social and historical relationships they have; the choices they make within the opportunities and constraints of historical and social circumstances (Bronfenbrenner and Morris, 2007). “It is, thus, vital understanding how historical legacies influence people’s views, attitudes, incentives, and decisions” (Michalopoulos and Papaioannou, 2020, p. 56) Sub-Saharan Africa has one of the highest rates of poverty (Anyanwu and Anyanwu, 2017) and inequality (Bhorat and Naidoo, 2018) in the world, leading to massive structural disparities resulting in limited and unequal access to educational, health, and protection services (Castells-Quintana et al., 2019). In addition, the region performs poorly on many health measures indicating that it is most affected by numerous health challenges associated with limited access to adequate health care (Wang et al., 2020). Sub-Saharan Africa is also one of the region’s most negatively affected by the devastating impact of climate change and global warming (Asongu et al., 2018), despite making the least contribution toward climatic changes at a regional and global level (Hickel, 2020; Ritchie et al., 2020). Finally, sub-Saharan Africa has high rates of different forms of violence, especially gender-based violence (Nabaggala et al., 2021). These challenges leave many adolescents with limited access to resilience-enabling resources and exposure to numerous risk factors that may impact their mental health outcomes.

Available data on poverty show that poverty decline in sub-Saharan Africa has been slow, especially when contrasted to other regions in the world (Anyanwu and Anyanwu, 2017; Asongu and Le Roux, 2019). Research on poverty levels suggests that 38.3% of the population (headcount ratio) in sub-Saharan Africa lives below the extreme poverty line of US$ 1.9 per day. Of concern is that according to the United Nations Development Programme [UNDP] (2017), “ten out of the 19 most unequal countries in the world are in sub-Saharan Africa” (Odusanya and Akinlo, 2020, p. 177).

When comparing health indicators for sub-Saharan Africa and global rates, profound inequalities emerge. As outlined by Wang et al. (2020), under-5 mortality rates in sub-Saharan Africa (74.1 deaths per 1,000) were just below double the global rate (37.1 deaths per 1,000); Life Expectancy at birth was 73.5 years at a global level but only 64.5 years at in sub-Saharan Africa (9 years less); and Healthy Life Expectancy was 6.1 years less in sub-Saharan Africa (57.4 years) than at the global level (63.5 years). In World Health Organization [WHO] (2015), sub-Saharan Africa accounted for 66% of global maternal deaths, with 1 in 31 women in sub-Saharan Africa at risk of maternal death compared to 1 in 4,300 women in developed countries. Sub-Saharan Africa has the highest rate of adolescent pregnancy in the world, with almost one in five (19.3%) adolescents in sub-Saharan Africa becoming pregnant (Kassa et al., 2018).

HIV/AIDS continues to be a leading cause of death in sub-Saharan Africa and accounts for 65% of new infections globally (James et al., 2018). HIV infections in sub-Saharan Africa disproportionately affect adolescents and young women (25% of new infections globally despite only representing 10% of the population), with 80% of new HIV infections amongst adolescents in the region occurring in girls between the ages of 15 and 19 years (Karim and Baxter, 2019). Women in sub-Saharan Africa bear the brunt of HIV in the region and are much more vulnerable than men due to various biological, behavioral, socioeconomic, cultural, and structural risks (Ramjee and Daniels, 2013). Stunting (a failure to grow in stature as a result of extended malnutrition) is common in sub-Saharan Africa, with a 36% prevalence rate (Watkins, 2016).

In relation to education, sub-Saharan Africa has an out-of-school rate of 18.8%, for children of primary school age, compared to the global rate of 8.2%, with females having a 5.1% higher rate than males in the region (United Nations Educational, Scientific and Cultural Organization [UNESCO], 2019). Access to education is uneven for girls and boys due to factors such as violence, poverty, early marriage, and negative cultural values leading to high levels of illiteracy for girls (Ombati and Ombati, 2012). For those in school, the quality of education they receive in sub-Saharan Africa is impacted by insecure working conditions for teachers (including low job security, wages, and motivation) and the superficial and inadequate level of teacher training (Lauwerier and Akkari, 2015).

Climate change and global warming are also concerning factors impacting sub-Saharan Africa. The global north (USA, Canada, Europe, Israel, Australia, New Zealand, and Japan) is responsible for 92% of the excess CO_2_ emissions and contribute 68% of the total proportion of CO_2_ emissions (Hickel, 2020), whereas the global south is disproportionately harmed by its consequences through atmospheric colonization (Hickel, 2017). Extreme weather events caused by climate change and global warming result in disruptions to food security, water and sanitation, education, and health sectors (Codjoe et al., 2020).

Violence and, in particular, gender-based violence, continue to be prevalent in sub-Saharan Africa, with 41.0 to 45.6% of women experiencing intimate partner violence (IPV) and 14% experiencing non-IPV (Muluneh et al., 2020; McClintock and Dulak, 2021; Nabaggala et al., 2021). Sub-Saharan Africa has much higher rates of violent crime (13%) than other regions (4%) (Corcoran and Stark, 2018).

Despite the numerous challenges adolescents in sub-Saharan Africa face, there is evidence of resilience amongst sub-Saharan African adolescents who are able to mobilize available protective resources to buffer against hardship and unexpectedly (given severe adversity) thrive (Ebersöhn, 2017; van Breda and Theron, 2018).

### 2.2. Resilience, adolescence, and mental health

Within contexts of adversity rooted within historical legacies, adolescent resilience needs to be understood. Resilience constitutes processes to navigate resources that sustain wellbeing and negotiate access to these in contextually appropriate ways (Ungar, 2011). Understanding adolescent access to the “essential ingredients for resilience” (Ungar, 2008, p. 45), such as relationships, powerful identity, power and control, social justice, access to material needs, a sense of belonging, and a sense of culture and roots–will enable us to explore their impact on mental health outcomes. Resilience is therefore embedded within the context in which an individual exists first, followed by the quality of the individual (Ungar, 2011). In line with this, resilience is understood as being linked to the concept of risk exposure and one’s ability to resist environmental risks or overcome adversity (Rutter, 2006). Here, risk exposure is not as important to consider as how individuals respond to or deal with these risks. Resilience is, therefore, not an individual trait but rather a complex interaction between the individual and their environment, which can change over time (Ungar, 2011).

Resilience, or an adolescent’s ability to navigate to the resources that can sustain their wellbeing, is seen as an important buffer against the negative impact of the challenges adolescents are confronted with (van Breda and Theron, 2018), especially in low-and-middle-income countries where they experience structural disadvantages and adverse living conditions. Research in South Africa has shown that despite experiencing high-risk factors due to poverty, individuals continue to adapt to adversity and express wellbeing (Ebersöhn, 2017). In their critical review of South African child and youth resilience studies, van Breda and Theron (2018) identified four resilience-enabling pathways, namely, personal (e.g., agency and adaptive meaning-making), relational (e.g., affective support and opportunities for growth development), structural (e.g., financial wellbeing and community safety), and spiritual/cultural (e.g., spiritual beliefs and cultural values).

Resilience for adolescents in sub-Saharan Africa should be understood from an ecological perspective and is particularly embedded within reciprocal supportive collectives, linked to impactful figures that adolescents are connected to Theron (2019). Resilience within this context is less about the individual and more about the complex interplay between the individual and the multiple social systems in which they exist (Wessells, 2021). Indeed, calls have been made to view resilience as a broader concept within a social-ecological model to include individual, interpersonal, and community contributions to resilience and move away from a narrow focus on the psychological and limited interpersonal understandings of resilience (Dulin et al., 2018).

This paper was guided by an ecological systems theoretical framework (Bronfenbrenner, 1979) using a resilience lens. Within this framework, development and resilience are understood to be a result of continuous interactions between the individual and their environment (Bronfenbrenner, 1979; Ungar, 2011; Overton, 2013). Within the context element of the theory is the understanding that children develop within nested structures with different levels of impact on their development, namely, the microsystem, mesosystem, exosystem, macrosystem, and chronosystem (Bronfenbrenner, 1977). The focus solely on the individual has been shown as limiting, and evidence suggests the need for a more nuanced and relational understanding of development and resilience (Lerner, 2006). Positive developmental outcomes in adverse conditions result from complex relational interactions between the individual, family, school, community, friends, service available, mass media, and culture (Ungar, 2011). Relationality emphasizes reciprocal, bi- or multi-directional, or circular causality (Overton, 2013). As individuals and context change, so do the factors associated with positive outcomes.

Ebersöhn’s (2012) proposed Relationship-Resourced Resilience echoes this by seeing resilience as a collective rather than an individual process whereby individuals with shared and persistent burdens connect (or flock) to access and share resources. Masten (2018) offers a scalable definition of resilience as “The capacity of a system to adapt successfully to significant challenges that threaten the function, viability, or development of the system.” (p. 5). For this paper, a resilience lens has been used to understand resilience outcomes for adolescents with mental health (self-esteem, depression and feeling safe) as mediator in selected LMICs in SSA and to explore the relationship between the predictors (the socio-demographic variables) and the output (resilience), with the mediators being feeling safe, depression, and self-esteem (which all link to mental health). Relentless and cumulative adversities in the sub-Saharan context means that a resilience lens is useful contextually as it focuses on adaptive functioning at the high end of the continuum of risk or adversity (Lerner et al., 2019), and high-risk children and youth (Masten, 2014).

## 3. Materials and methods

### 3.1. Design

A cross-sectional comparative case study research design was employed. Comparative case study research seeks to enhance knowledge about society as a process by exploring the differences and similarities among large macrosocial units such as countries (Ragin, 2014). The study compared (across countries) the case of adolescent mental health of adolescents in low-middle-income countries within sub-Saharan Africa. The countries were selected based on the work done by a regional non-governmental organization called the Regional Psychosocial Support Initiative (REPSSI). REPSSI has worked in the Eastern and Southern African Region since 2002 and has established itself as the leading sub-Saharan African psychosocial support organization. REPSSI contributes to the growing body of evidence for psychosocial and mental wellbeing as a critical enabler of social, health and education outcomes for children, adolescents, and youth, provides evidence-based technical assistance and leads advocacy for sustained psychosocial support mainstreaming into social services, health and education programmes and services. It focuses on girls, boys, youth, families, and communities in SSA to ensure they live with hope, dignity, and happiness. REPSSI works across thirteen low-middle-income countries in SSA: Angola, Namibia, Zambia, Botswana, Kenya, Mozambique, Malawi, Zimbabwe, Lesotho, Tanzania, Eswatini, South Africa, and Uganda. REPSSI is implementing several psychosocial interventions for adolescents across all the countries where they have a presence. The first author works with REPSSI and oversees its research work. Countries, where REPSSI was implementing interventions and collecting data, were selected.

### 3.2. Sampling and sample

Purposive sampling was used in the present study, whereby a sample of adolescents involved in REPSSI interventions was invited to participate before the start of the intervention. An attempt was made to obtain data from 20% of adolescents participating in REPSSI interventions. In-country teams were asked to select a group of adolescents to collect data from, and once the target per country was reached, data collection was suspended for that group or country. Data were collected from 3,312 of the 15,979 adolescents who accessed REPSSI interventions, constituting a 21% coverage. The inclusion criteria for respondents were that they were part of REPSSI interventions, consented to be part of the present study, and were over 12 years of age. Respondents with unique characteristics such as gender (with the aim of an equal number of female and male respondents), location (with the aim of an equal number of respondents by intervention sites), and age (focusing on respondents between the ages of 12 and 20) were purposively selected. A total of 3,312 responses were collected across nine low-or-middle-income countries sub-Saharan Africa countries. The sample included 1,818 female respondents (55.0%) and 1,494 male respondents (45.0%). Data were collected from respondents in Angola (385, 11.6%), Eswatini (128, 3.9%), Kenya (390, 11.8%), Lesotho (349, 10.5%), Mozambique (478, 14.4%), Namibia (296, 8.9%), South Africa (771, 23.3%), Uganda (201, 6.1%), and Zambia (314, 9.5%). The ages of respondents ranged between 12 and 20, with a mean age of 14.58 years (SD = 1.82).

### 3.3. Data collection

Quantitative primary data were collected through a survey administered directly to respondents over the period of March and October 2020. REPSSI staff with experience in research processes in each country were trained in the tool and data collection processes. Data collectors with some experience in data collection and knowledge of mental health were recruited and trained in each country in line with the training guide. Given that data were collected from young people, younger data collectors were given preference. A training manual was created to standardize the training provided across countries and increase quality control. In addition, the REPSSI regional Monitoring and Evaluation officer provided ongoing support to each country during the training and data collection process. Training took place in person, although COVID-19 influenced how this happened by ensuring that all protocols were implemented to protect data collectors.

The questionnaire was translated into local languages by individuals with experience in translation who were fluent in both languages. The tools were translated into Portuguese (for Angola and Mozambique), Herero (for Namibia), and Swahili (for Eswatini). Given the limited resources, this process was limited to forward translations by single bilingual individuals. A limitation of this approach is that it may result in lower levels of reliability and validity of the data collected (Abubakar et al., 2013). The use of inter-item reliability tests and confirmatory factor analysis, which were undertaken for each scale, confirmed that despite the limitations of the translations, the psychometric characteristics of the scales were good (Swami and Barron, 2019).

Data collection was done close to the start of the implementation of the intervention in the country. Before the start of the interventions with adolescents, those for whom consent was obtained were contacted by the data collector to arrange for data collection. Data were collected in person using tablets. Once all responses were collected, data were uploaded into a cloud-based server which used data encryption in transit, at rest, and on all backups. Only the first author had complete access to all the data, including identifying data. Confidentiality was maintained throughout all study procedures by storing locator information separately from respondent data. No identifying data were extracted from the database for analysis.

### 3.4. Measures

#### 3.4.1. Demographic variables

Respondents’ socio-demographic information collected included information on age (continuous variable), gender (binary variable: male, female), country (nominal variable: open-ended), school attendance (binary variable: yes, no), type of dwelling (nominal variable: house made of brick, house made of traditional materials, house made of steel sheets on its own plot, house made of steel sheets in a backyard, block of flats. a children’s home or shelter, on the street, other), access to water and electricity at home (binary variable: yes, no), whether they are attending school (binary variable: yes, no), days hungry in the last week (continuous variable measured by framing the question as “how many days did you go to sleep hungry in the previous week?”), parental loss (two binary variables on being single or double orphaned: yes, no), family situation (two binary questions about looking after sick people or younger children at home: yes, no).

#### 3.4.2. Resilience scales

The Child and Youth Resilience Measure-Revised (Jefferies et al., 2019) was used to measure the resources available to individuals that may bolster their resilience. The scale provides an overall resilience score, a caregiver resilience score (which includes items associated with relationships with either a primary caregiver of family), and a personal resilience score (which includes intrapersonal and interpersonal items), with the latter two linked as they depend on the social ecologies of the individual to reinforce resilience (Jefferies et al., 2019). The scale consists of 17 items which are rated on a 5-point Likert scale from *not at all* (1) to *a lot* (5). All 17 items were summed to compute a total index of resilience which yielded a Cronbach’s alpha equal to 0.83. Ten items were summed to compute an index of personal resilience, which yielded a Cronbach’s alpha equal to 0.74. Seven items were summed to compute an index of caregiver resilience which yielded a Cronbach’s alpha equal to 0.71. The Child and Youth Resilience Measure (CYRM) was developed as part of the International Resilience Project at the Resilience Research Centre in 14 communities worldwide, including the Gambia, Tanzania, and South Africa (Ungar and Liebenberg, 2011). Our reliability analysis confirms the two-factor structure found by Jefferies et al. (2019), with 10 items belonging to the personal resilience scale and 7 items belonging to the caregiver resilience scale. The items and which scales they belong to are laid out in Table 5 of Jefferies et al. (2019).

#### 3.4.3. Depression scale

The Patient Health Questionnaire-9 (PHQ-9) is a self-administered diagnostic screening tool for assessing and monitoring depression severity (Kroenke et al., 2001; Blackwell and McDermott, 2014). The PHQ-9 was developed based on the nine criteria upon which the diagnosis of depressive disorders in the Diagnostic Statistical Manual – IV (DSM-IV) is based (Kroenke et al., 2001). The scale consists of nine items rated on a 4-point Likert scale from *not at all* (1) to *nearly every day* (4). All items were summed to compute a total index of depression which yielded a Cronbach’s alpha equal to 0.75. The PHQ-9 is useful, reliable, and valid in similar contexts too where we will use it, such as South Africa (Bhana et al., 2015; Aggarwal et al., 2017; Baron et al., 2017), Tanzania (Nolan et al., 2018; Smith Fawzi et al., 2019), Malawi (Udedi et al., 2019), Ghana (Anum et al., 2019), and Ethiopia (Gelaye et al., 2013).

#### 3.4.4. Self-esteem scale

The Rosenberg Self-esteem Scale is a 10-item scale that measures global self-worth by measuring positive and negative feelings about the self (Rosenberg, 1965). Global self-worth is defined as one’s overall sense of worthiness as a person (Rosenberg, 1965). The scale consists of ten items rated on a 5-point Likert scale from *not at all* (1) to *a lot* (5). All items were summed to compute a total index of self-esteem which yielded a Cronbach’s alpha equal to 0.70. The “Rosenberg Self-esteem Scale” (Rosenberg, 2006, p. 61) has been administered to over 16,000 people in over 50 countries, including Botswana, South Africa, and Zimbabwe and found to be a useful measure of self-esteem with a Cronbach’s alpha value above 0.70 in these countries (Schmitt and Allik, 2005).

#### 3.4.5. Feelings of safety scale

In the questionnaire there was a self-developed section titled “feeling safe” which consisted of four ordinal Likert-scale items with response options “not at all,” “a little,” “somewhat,” and “quite a bit” to statements “I feel safe at home,” “I feel safe at school,” “I feel safe in my community” and “I don’t feel safe.” As these were self-developed, the reliability of the scale was established as follows. For establishing reliability, Cronbach’s alpha is the most widely used statistic; however, it is inappropriately applied to scales with few items as scales with few items are vulnerable to underestimation due to the property that Cronbach’s alpha value increases as the number of items on the scale increases (Pallant, 2020; Robertson and Evans, 2020). The recommendation is that inter-item correlations are a more appropriate measure of scale reliability, with there being different recommendations ranging from 0.1 (Pallant, 2020) and 0.3 (Hajjar, 2018) or higher acceptable, with values between 0.2 and 0.4 being optimal (Robertson and Evans, 2020). After reverse-scoring the negatively phrased item “I don’t feel safe,” all correlations were statistically significant (*p* < 0.001) and fell within the recommended values. The *p*-values are omitted from Table 1 as all *p* < 0.001, indicating all correlations are statistically significant. As expected, all correlations are positive, showing that feeling safe at home, at school and in my community, as significantly and positively correlation, however, also as expected, the item “I don’t feel safe” is statistically significantly negatively associated with the other positively phrased items.

**TABLE 1:** Spearman correlations of feeling safe items.

Item	1.	2.	3.	4.
1. I feel safe at home	1.000	0.310	0.304	−0.135
2. I feel safe at school	0.310	1.000	0.427	−0.111
3. I feel safe in my community	0.304	0.427	1.000	−0.169
4. I don’t feel safe	−0.135	−0.111	−0.169	1.000

### 3.5. Respondents’ demographic characteristics

Almost all respondents (3,182, 96.1%) indicated they were in school. Earlier, in the literature review, we mentioned that SSA has an out-of-school rate of 18.8% for children of primary school age, meaning the in-school rate is 81.2%, whereas our results indicated that 96.1% of respondents indicated that they were in school. It should be noted that, in the projects that REPSSI are involved in, most of the access to children is done through schools; hence the high percentage reported of those in school. Of those that indicated that they were not in school (130, 3.9%), most said they were not in school because they did not have enough money (61, 46.9%), dropped out (28, 21.5%), got pregnant (13, 10.0%), or because they lost interest in school (11, 8.5%). Most respondents indicated that they lived in a house made of brick (1,274, 38.5%), followed by a house made of traditional materials (780, 23.6%), then a house made of steel sheets on its own plot (474, 14.3%), a block of flats (404, 12.2%), and a house made of steel sheets in a back yard (339, 10.2%). Almost none of the respondents reported living in a children’s home or shelter (8, 0.2%) or on the street (5, 0.2%). Approximately half (1,546, 46.7%) of the respondents indicated that they had a tap with running water in their house, while just over two-thirds (2,284, 69.0%) indicated that they had electricity connected to their house. Approximately one-fifth (705, 21.3%) of respondents indicated that they went to sleep hungry one or more days in the past week, with 12.1% (400) saying that they went two or more days hungry in the last week. The mean number of days respondents went to bed hungry in the last week was 0.42 (SD = 0.98).

### 3.6. Data analysis

The primary analysis focused on the description of the resilience status of respondents and also explored between-country differences. Pearson’s Chi-square test (χ^2^), also known as a Chi-square test for independence or test of association, was used to determine whether there is a significant relationship between two categorical or nominal variables. Path and mediation analyses were conducted which allows one to examine the direct and indirect effects of one variable on another. They can help one understand the underlying mechanisms and pathways through which variables are related. Significance testing was undertaken using a 5% level of significance; thus, *p* < 0.05 indicates statistical significance. However, the use of *p*-values has received much criticism over the years (Nuzzo, 2014; Betensky, 2019), with one major criticism being its dependence on sample size (i.e., one might find a statistically significant result purely due to large sample size). Accordingly, researchers advocate for considering not only considering a low *p*-value but coupling it with an effect size (*d*) that is considered “medium” or “large” (Goodman et al., 2019; Halsey, 2019), as effect size is independent of the sample size. Thus, in the present study, only the results where *p* < 0.05 and *d* is moderate or higher, will be considered. Note that, *d* is typically used to denote the well-known effect size Cohen’s *d*, which is associated with the parametric Student’s *t*-test, and for the well-known *t*-test, 0.5 < = *d* < 0.8 (medium) and *d* > = 0.8 (large). However, in the present study, *d* will be used to denote the effect size for all statistics considered with 0.3 < = *d* < 0.5 (medium) and *d* > = 0.5 (large) being cut-off points for χ^2^ tests (Kotrlik et al., 2011) and for any regression-type analysis (Nieminen, 2022) such as path and mediation analyses where the standardized regression coefficients represent the effect size. Thus, only results with *p* < 0.05 and *d* > = 0.3 will be reported on in the present study. STATA v14 was used for the descriptive and Chi-square statistics, whereas AMOS v28 was used for the path and mediation analyses.

### 3.7. Limitations

The current study employed a cross-sectional design, whereby data were collected from participants at a single point in time. Unlike longitudinal studies that track the same group of participants over an extended period, cross-sectional studies do not allow for establishing causal relationships. Therefore, the data collected in the current study does not provide direct support for causality. In the current study, the terms “direct effect” and “indirect effect” are used within the context of mediational analyses. It is important to note that these terms are not utilized to establish or imply causality. Rather, they are employed as standard language within mediational analyses to describe the relationships and pathways observed between variables.

Data were collected over the period of March and October 2020. By March 2020, since the World Health Organization (WHO) had declared COVID-19 a “Public Health Emergency of International Concern” on 30 January 2020 and a “pandemic” (p. 2) on 11 March 2020 (Lane et al., 2021, p. 2), all the countries considered in the current study already had introduced some policies and containment measures during (or before) the month of March 2020; details and timelines can be found at United Nations [UN] (2022) for Angolo, Nchanji and Lutomia (2021) for Eswatini, Lesotho, and Kenya, Lane et al. (2021) for Mozambique and Namibia, and Haider et al. (2020) for South Africa, Uganda and Zambia. As all countries were aware of the global public health emergency status and had implemented some level of response measures to address the pandemic’s impact by the time of data collection, although we cannot measure how the COVID-19 pandemic restrictions may have impacted the adolescents’ responses, the adolescents’ experiences were captured in all nine counties under the shared knowledge and awareness of COVID-19’s severity and implications.

The measure on feelings of safety was created by the team to assess how safe adolescents felt in different contexts and how their feelings of safety in different contexts correlate to wellbeing. This may have resulted in a very broad measure of safety. It may have strengthened the research to use a specific scale to measure feelings of safety, such as the Neuroception of Psychological Safety Scale (NPSS) (Morton et al., 2022).

The study focuses on adolescents in SSA, which has unique social, economic, and environmental characteristics. The challenges and risk factors mentioned, such as poverty, inequality, climate change and global warming impacts, and high rates of violence (especially gender-based violence), are specific to this region. Therefore, the findings may not be applicable to adolescents in other regions or countries with different contextual factors.

## 4. Results

Due to the large number of countries and the vast number of variables considered in the present study, only the result results are reported on and considered in this section. As this was a cross-country analysis, first, differences between the countries are considered. When exploring differences between countries in overall resilience [χ^2^(8) = 440.604, *d* = 0.365, *p* < 0.001], personal resilience [χ^2^(8) = 350.137, *d* = 0.325, *p* < 0.001], and caregiver resilience [χ^2^(8) = 379.851, *d* = 0.339, *p <* 0.001] levels, significant differences were found. Angola had significantly lower overall resilience levels (*Mdn* = 61, *IQR =* 15) than all other countries. Lesotho respondents had significantly higher overall resilience levels (*Mdn* = 74, *IQR =* 12) than Angola (*Mdn* = 61, *IQR =* 15), Eswatini (*Mdn* = 65, *IQR =* 22.5), Mozambique (*Mdn* = 65, *IQR =* 12), Uganda (*Mdn* = 66, *IQR =* 15), and Kenya (*Mdn* = 72, *IQR =* 17). As with overall resilience, Angola had significantly lower personal resilience levels (*Mdn* = 35, *IQR =* 12) than all other countries. Respondents from Lesotho had significantly higher personal resilience levels (*Mdn* = 44, *IQR =* 9) than Angola (*Mdn* = 35, *IQR =* 12), Eswatini (*Mdn* = 39, *IQR =* 14), Mozambique (*Mdn* = 39, *IQR =* 8), Uganda (*Mdn* = 40, *IQR =* 9), South Africa (*Mdn* = 42, *IQR =* 8), and Kenya (*Mdn* = 42, *IQR =* 9). Regarding caregiver resilience, both Angola (*Mdn* = 27, *IQR =* 6) and Mozambique (*Mdn* = 27, *IQR =* 6) had significantly lower levels than Kenya (*Mdn* = 31, *IQR =* 9), Lesotho (*Mdn* = 31, *IQR =* 6), Namibia (*Mdn* = 31, *IQR =* 6), South Africa (*Mdn* = 31, *IQR =* 6), Zambia (*Mdn* = 31, *IQR =* 8), and Uganda (*Mdn* = 28, *IQR =* 7).

Mozambique had the highest proportion of respondents reporting that they had access to running water in their homes (80.5%), followed by South Africa (75.6%), Namibia (49.3%), and Zambia (40.1%), and these differences were significant [χ^2^(8) = 902.893, *d =* 0.522, *p* < 0.001]. Countries with the lowest proportion of respondents reporting that they had access to running water in their homes were Uganda (9%), Kenya (15.6%), and Lesotho (21.8%). Similarly, a significant difference was found between the proportion of respondents who had access to electricity in their homes across countries [χ^2^(8) = 850.521, *d* = 0.507, *p* < 0.001]. Again, almost all countries showed significant differences in home access to electricity, except between Angola and Eswatini, Angola and Kenya, Eswatini and Kenya, Lesotho and Zambia, Namibia and Zambia, and Lesotho and Namibia. Mozambique (98.3%) and South Africa (89.8%) had the highest proportion of respondents reporting electricity in their homes, with the lowest percentage being Uganda (11.4%).

A mediation analysis was conducted to investigate the relationship between the predictors (the socio-demographic variables) and the output (resilience), with the mediators being feeling safe, depression, and self-esteem (which all link to mental health). By examining the indirect effects of the socio-demographic variables on resilience through the mediators, mediation analysis provides insights into the extent to which feeling safe, self-esteem and depression (i.e., elements contributing to mental health) contribute to the relationship between these predictors and the ultimate outcome of resilience. Mediation analysis allows one to examine the direct and indirect effects of one variable on another. For the mediation analysis, when considering all countries collectively, the only significant results (when considering both direct and indirect effects) is that feeling safe has a significant direct effect on overall resilience (*β* = 1.239, *SE* = 0.067, *d* = 0.343, *p* = 0.003) and on personal resilience (*β* = 0.758, *SE* = 0.043, *d* = 0.331, *p* = 0.005). Note that *β*, *SE*, *d* and *p* refer to the regression coefficient, the standard error, the effect size and the *p*-values, respectively. Thus, the stronger the sense of feeling safe, the higher one’s overall and personal resilience. Since “feeling safe” is the only variable that had a significant effect on resilience, and since the Chi-square test indicated that there are some differences between the countries, a path analysis was conducted for each country to explore the effect of feeling safe per country, as the effect of feeling safe on resilience wanted further exploration. It is interesting to note (see Table 2) that feeling safe has a direct effect on overall and personal resilience for all countries; however, for caregiver resilience, the direct effect is significant for all countries except for South Africa and Mozambique.

**TABLE 2:** Result of path analysis.

Country (*n*, %)	Resilience	β	*SE*	*d*	*p*	Significant
Angola (385, 11.6%)	Overall	1.871	0.144	0.577	0.006	Yes
	Personal	1.137	0.104	0.497	0.005	Yes
	Caregiver	0.734	0.068	0.503	0.005	Yes
Eswatini (128, 3.9%)	Overall	2.342	0.414	0.503	0.007	Yes
	Personal	1.247	0.245	0.469	0.006	Yes
	Caregiver	1.094	0.195	0.490	0.007	Yes
Kenya (390, 11.8%)	Overall	3.279	0.266	0.590	0.003	Yes
	Personal	1.655	0.143	0.541	0.004	Yes
	Caregiver	1.624	0.142	0.566	0.002	Yes
Lesotho (349, 10.5%)	Overall	1.655	0.204	0.436	0.004	Yes
	Personal	0.996	0.136	0.408	0.003	Yes
	Caregiver	0.659	0.103	0.350	0.002	Yes
Mozambique (478, 14.4%)	Overall	1.101	0.118	0.402	0.003	Yes
	Personal	0.722	0.085	0.395	0.004	Yes
	Caregiver	0.378	0.055	0.282	0.005	No
Namibia (296, 8.9%)	Overall	1.445	0.211	0.453	0.006	Yes
	Personal	0.873	0.128	0.441	0.005	Yes
	Caregiver	0.572	0.093	0.399	0.005	Yes
South Africa (771, 23.3%)	Overall	0.930	0.113	0.313	0.003	Yes
	Personal	0.640	0.078	0.311	0.004	Yes
	Caregiver	0.290	0.057	0.203	0.003	No
Uganda (201, 6.1%)	Overall	1.432	0.351	0.476	0.007	Yes
	Personal	0.803	0.216	0.437	0.005	Yes
	Caregiver	0.629	0.152	0.403	0.004	Yes
Zambia (314, 9.5%)	Overall	2.009	0.229	0.452	0.003	Yes
	Personal	1.234	0.148	0.448	0.003	Yes
	Caregiver	0.775	0.128	0.343	0.003	Yes

## 5. Discussion

We start the discussion by considering the results of the mediators (depression, self-esteem and feeling safe). In this sample, depression levels were found to be relatively low, with the mean score being 5.63 out of a possible range of between 0 and 27. Only 46 respondents (1.4%) had scores in the highest third range (above 18), 16.7% scored between 9 and 18, and the majority of respondents (81.9%) scored in the lowest third range (below 9). Suggested best cut-off points for detecting major depressive disorder using the PHQ-9 vary between 8 (Haddad et al., 2013; Urtasun et al., 2019) and 10 (Kiely and Butterworth, 2015; Costantini et al., 2021). Prevalence rates of depression within the present study’s sample were 15% using a cut-off of 10 or 23% using a cut-off of 8. Available literature on prevalence of depression is limited in that the regional coverage is highly variable, and methodological issues (varying definitions, non-generalizable sample sizes, and a lack of standard indicators) prevent amalgamation across countries (Baxter et al., 2013). Available literature on prevalence of depression among adolescents in sub-Saharan Africa varies between 9.3 and 21.8% (Cortina et al., 2012; Nalugya-Sserunjogi et al., 2016; Aggarwal et al., 2017; Ajaero et al., 2018; Kyohangirwe et al., 2020; Quarshie et al., 2020). Given the prevalence rates of depression among adolescents in sub-Saharan Africa found by other studies, a more conservative cut-off of 10 for depression may be more suitable for this sample. The present study’s prevalence rate of 15.0% for depression concurs with that in existing literature, namely, of depression prevalence rates of between 9.0 and 22.0% amongst adolescents in sub-Saharan Africa. The relatively low depression prevalence rate amongst this sample may indicate that adolescents in SSA are managing to cope with their contextual challenges and that these may not be impacting their levels of depression. On the other hand, an argument could be made that 15 out of 100 adolescents having depression in a non-clinical sample is of concern and should be addressed. Further research could explore how depression presents itself within this sample and ensure that the existing definitions and diagnoses of depression are culturally and contextually sensitive. In addition, further research could explore other ways in which adolescents’ mental health in SSA may be affected (beyond depression). Interventions for adolescents in SSA could focus on how to increase access to protective factors against depression. Furthermore, interventions could ensure that those that do present with depression receive appropriate differentiated interventions specific to their mental health challenges.

Self-esteem levels of respondents could be described as moderate to good, with the mean score being 29.4 out of a possible range of between 10 and 40. Only 11 respondents (0.8%) scored in the lower third range (below 20), while 64.2% scored between 21 and 30, and 35.0% scored in the highest third range (above 30). Using the median of 25 to dichotomize the scores (of the possible range for the scale), for the present study, 13.6% would be categorized as having low self-esteem (below 25) while 86.4% would be categorized as having high self-esteem (above 25). Researchers have reported divergent results in relation to self-esteem, with high levels of self-esteem found in 90.9% of university medical students in Nigeria (Egwurugwu et al., 2017), 50.9% of young people living with HIV in rural South Africa (Filiatreau et al., 2020), 42.9% of 12 to 24-year-olds living in rural South Africa (Filiatreau et al., 2021), 39.7% of 10 to 14-year-old primary school children in Uganda (Kemigisha et al., 2018), and 13% (32% moderate) of university students in Nigeria (Coker et al., 2019). The present study found a much higher proportion of adolescents with high levels of self-esteem (86.4%) than existing literature (with the exception of the Nigeria study with medical students), which may be linked to differences in the samples. Comparatively high levels of self-esteem may be indicative of the particular sample (i) having sufficient protective resources that support the positive development of adolescent self-esteem, or (ii) the risk factors that constrain the development of positive self-esteem development are limited. Further research exploring notions of the “self,” how adolescents in sub-Saharan Africa feel about their own self-worth, and how this differs from existing literature and knowledge of self-esteem would be valuable.

For safety, the possible score ranges from 0 to 16 (as there were four questions with response options coded 0 to 4) with the mean being 10.87. Almost a third of respondents (29.3%) scored in the lower third range (10 or lower), while almost half (48.1%) scored between 10 and 12, and 22.6% scored in the highest third range (12 or higher). The fact that a higher percentage is in the lower third range than the higher third range shows feelings of feeling unsafe. Many studies have linked feeling unsafe to mental health problems (Mori et al., 2021; Fossum et al., 2023) and this is why the variable relating to feelings of safety was selected as a mediating variable. The results showed that the safer you feel, the higher your resilience (overall and personal for all countries) and caregiver (for all countries except two). In addition, feeling safe was the only variable to have a direct effect on resilience out of the three mediating variables, namely, depression, self-esteem and feeling safety.

Now turning to socio-demographic variables, in the current study, gender did not have a significant direct or indirect effect on resilience. Existing literature on gender differences in relation to resilience is mixed in that some have found higher levels of resilience in females (Sun and Stewart, 2007; Newsome et al., 2016), and others found higher levels of resilience in males (Boardman et al., 2008; Erdogan et al., 2015; Fallon et al., 2020; Yalcin-Siedentopf et al., 2021). Despite these differences in literature, the common factor is that existing literature seems to point to there being a gendered dimension to resilience. However, in contrast to existing knowledge on the gendered nature of resilience, in the present study, gender did not have a significant direct or indirect effect on overall, personal, or caregiver resilience. Of interest is that a study in a similar context (Malawi and South Africa) but with children younger than those in the current study (Macedo et al., 2018) also found no gender differences in relation to levels of resilience. The absence of gender differences in resilience, self-esteem, or depression within the present study may indicate that adolescent girls and boys are similar in terms of their access to resilience-enabling resources and their levels of depression and self-esteem. From a research perspective, a more detailed exploration of how female and male adolescents may differ in their presentation of depression, their notions of self-esteem, and their resilience may be required. For practitioners, interventions that focus on adolescents’ access to resilience-enabling resources, reduce depression, or enhance self-esteem may not require gender-specific content.

When turning to the predictors, it was interesting to note that there were no direct or indirect significant effects between the predictors (socio-economic variables) with the three mental health mediators (depression, self-esteem and safety) and the output (resilience). These results seem to be in contrast with some literature but also in agreement with findings from other literature. Take age, for example. Some research has found that being younger was associated with lower levels of depression (Thapar et al., 2012; Gardner and Lambert, 2019; Khesht-Masjedi et al., 2019). On the other hand, the results of the present study, particularly there being no significant direct effect between age and self-esteem, concur with other existing research which has found that self-esteem levels remain stable during adolescence (Orth et al., 2018) and decrease in older adolescents and increase from adolescence to middle adulthood (Trzesniewski et al., 2013; Orth and Robins, 2014). On the other hand, Gardner and Lambert (2019) have found that older adolescents had lower levels of self-esteem, which contradicts the present study, which found that levels of self-esteem did not differ significantly depending on age. Of course, there were many other socio-economic variables that, for the current study, were shown to have no significant direct or indirect effect on mental health outcomes and resilience, which concurs and contradicts many findings in the literature, therefore, studies such as these are valuable in exploring relationships such as these.

### 5.1. Enabling resilience of adolescents in sub-Saharan Africa

The current comparative study contributes to the literature on the links between mental health (mediator) and resilience (outcome) for adolescents in sub-Saharan Africa. The paper contributes to the existing literature by outlining the centrality of crafting safe spaces in which adolescents may live as they navigate resilience.

At a **macrosystems** level, efforts to address the numerous challenges adolescents in sub-Saharan Africa experience, such as poverty, inequality, educational shortcomings, health crises, and violence, need to be enhanced. In addition, our results associating being in school with better levels of resilience and mental health provide support for the need for sub-Saharan Africa to critically address the high rate of out-of-school population and the low education participation rate (United Nations Educational, Scientific and Cultural Organization [UNESCO], 2019). Given the potential protective resources schools are in moderating the negative impact of poverty (Ashley-Cooper et al., 2019) and positive mental health outcomes (Aldridge and McChesney, 2018), ensuring that children are in school and remain in school could be a priority for sub-Saharan Africa. Access to mental health services for adolescents needs to be improved in sub-Saharan Africa; access to mental health services is severely limited for adolescents who require in-patient treatment in sub-Saharan Africa as the availability of such services is decreasing and scarce (Owen et al., 2016; Dlamini and Shongwe, 2019). At the same time, the scarcity of mental health services in sub-Saharan Africa makes research into the socio-ecological pathways to better mental health outcomes, such as the present study, important.

At an **exosystem level**, efforts need to be made to ensure that children and adolescents have sufficient access to nutritious food and are not affected by hunger. Positive adolescent development and mental health rely on adolescents’ ability to access nutritious food, and it is the responsibility of families, communities, and governments to provide such access. The present study contributes to existing knowledge in supporting the link between feeling safe in communities and mental health outcomes (Traoré et al., 2020; Pearson et al., 2021). Creating opportunities for adolescents to feel safe within their broader environment can therefore enhance their mental health outcomes and be resilience-enabling (Reich et al., 2017). In fact, the results suggest that feeling safe is a key factor in adolescent mental health and resilience.

At a **mesosystem level**, feeling safe at school and at home was associated with higher levels of resilience, lower levels of depression, and higher levels of self-esteem. Safety and protection of adolescents need to be prioritized in sub-Saharan Africa. Schools can provide access to resilience-promoting resources (Ebersöhn et al., 2015; Jefferies and Theron, 2017), which improve outcomes for adolescents (Ungar et al., 2019), including improved wellbeing and better mental health outcomes (Moore et al., 2018). Schools can be places to address resilience in contextually relevant ways, pre-empt risks, and advocate for systemic changes that enhance adolescent wellbeing (Theron, 2016). The present study indicates that fostering positive, caring relationships between adolescents and their peers could be a useful strategy to protect adolescents against poor mental health outcomes.

As adolescents move from early to late adolescence, peer relationships increasingly influence their behavior (De Goede, 2009), and therefore these relationships could be a focus of interventions to protect adolescents’ mental health. However, peer interactions are also found to increase risk-taking (Knoll et al., 2015) and problem behaviors (Dishion and Tipsord, 2011) in adolescents, Therefore, enhancing positive peer relationships could be a key intervention strategy in terms of influencing better mental health outcomes for adolescents. Developing adolescents’ skills to create and maintain positive, encouraging, supportive, trusting, and connected friendships amongst each other could enhance their mental health outcomes (Woods-Jaeger et al., 2020; Cutuli et al., 2021). The present study confirms that peer support can be protective against depression (Roach, 2018) for adolescents in sub-Saharan Africa. The importance of positive relationships as protective resources for adolescents supports Ebersöhn’s (2012) proposed Relationship-Resourced Resilience concept, which highlights the collective rather than the individual process of resilience and the potential to leverage flocking as a mechanism to access and share resources.

At a **microsystem level**, adolescents who experience parental loss are in particular need of interventions that support them and enhance their access to resilience-enabling resources. The present study adds to existing literature which indicates that parental loss impacts resilience (Onkari and Itagi, 2019; Walsh, 2019), especially for adolescents (Kennedy et al., 2018). Ensuring that adolescents who have experienced parental loss are in line for additional support and services is warranted. Looking after younger children or sick people at home was found to be protective against poor mental health outcomes for adolescents in the present study, which contradicts existing literature (Cree, 2003; Cluver et al., 2012; De Roos et al., 2017). A more in-depth exploration of the associations between being a young career and mental health outcomes for adolescents in sub-Saharan Africa is recommended. The function of being a young career as a protective relational mechanism (Ebersöhn, 2012; Ungar, 2012) within sub-Saharan Africa could be explored further.

At an **individual level**, older adolescents seem to require additional attention in terms of their mental health. As adolescents age, their access to protective resources may be diminished during a time in which they could actually be enhanced. Their developmental need for independence and the search for peer and sexual or intimate relationships (Zani and Cicognani, 2020) may invertedly reduce their access to the protection offered by caregivers and school and increase their risk of poor mental health outcomes. Caregiver emotional engagement and support for autonomy can influence sexual agency for adolescents and support the development of positive intimate relationships (Klein et al., 2018). Interventions which encourage the continued, but developmentally appropriate involvement of caregivers and schools in supporting adolescents would be beneficial for adolescents in sub-Saharan Africa.

Finally, the **chronosystem** also needs to be considered, whereby the socio-historical context, the events adolescents experience at different ages, what age they are, the relationships they have access to, and the decisions they make within these contexts (Bronfenbrenner and Morris, 2007) influence both their feelings of safety and their access to resources that could enhance safety and therefore resilience.

The results of the present study indicate the necessity to foreground youth development intervention focus on collaboration across socio-ecological systems with the aim to create spaces where adolescents may experience feeling safe. We have argued that the Sub-Saharan African context compromises potential to “feel safe” as challenged systems interact. At macrosystem level poverty, inequality, educational limitations, health crises, and violence predict high instances of everyday crime, abuse and violence. The exosystem is characterized by constraints to everyday opportunities to experience safety in the community, as is the case on the mesosystem within schools, homes and amongst acquaintances. At the microsystem level the potential to feel unsafe is exacerbated by high prevalence of parental loss and demands on adolescents to act as caregivers to others in a household.

The present study supports existing research which highlights the interaction between individual, relational, and contextual resources to have a relationship with outcomes (Liebenberg, 2020). Focusing only on the individual is insufficient within this context and broader approaches across various socio-ecological systems would be more effective. This is especially important for adolescents who experience rapid developmental changes (Gibbs, 2019) and are at an increased risk for social-emotional disorders (Rapee et al., 2019). The present study provides further evidence of the socio-ecological understanding of resilience as embedded within the availability of resilience-enabling resources within their ecologies and adolescents’ ability to navigate their way to these resources in meaningful ways (Ungar, 2008, Ungar, 2011).

Youth development intervention strategies may benefit from leveraging socio-cultural and contextual practices that enable adolescents to feel safe. In this regard intervention strategies may include partnerships with adolescents at community-level to establish conditions in which adolescents feel safe such as street-committees to identify unsafe spaces, physical after-school leisure spaces (Baldwin, 2011), mapping systemic role-players (guardians, peers, police, mentors) who establish and maintain such spaces of safety (Levy et al., 2020), involving adolescents in the process of creating safe spaces (Chomat et al., 2019; van der Westhuizen et al., 2023) and deliberating how to resource and support new initiatives, and/or maintain existing mechanisms that enable feelings of safety amongst adolescents.

## 6. Conclusion

Given the scarcity of available literature on how mental health outcomes of adolescents in sub-Saharan Africa (the mediators) could be enhanced, this cross-country comparison adds valuable insight into the similarities and differences across the regions. The study found that feeling safe was the only variable to have a significant direct effect on resilience and that none of the indirect effects were significant. This comparative study supports the need to utilize a contextually informed approach to understanding resilience (Höltge et al., 2021) and addressing adolescent mental health needs (Ssewamala and Bahar, 2022) in SSA. The comparative differences found across countries in the present study further support the concept that resilience is context-specific (Herrman et al., 2011), requiring an understanding of the locally available cultural resources and individual thoughts, feelings, and behaviors of adolescents (Ungar and Theron, 2020). Different countries will require different approaches to enhancing access to resilience-enabling resources for better mental health outcomes for adolescents. Interventions could tap into existing collective psychosocial support practices to address the needs of adolescents within SSA communities (Ebersöhn et al., 2018). Further research is indicated to understand the country-level difference between Mozambique and South Africa, and other participating countries with regard to the direct effect of caregiver resilience and feeling safe.

## 7. Recommendations for future research

As the current study included a self-developed safety scale, the reliability of which was established, additional research could be conducted on the use of this safety scale or on other aspects of safety, such as psychological safety. In psychologically secure environments, individuals experience a sense of acceptance and worth, leading to increased trust, collaboration, and open communication. The Polyvagal Theory (PVT), which provides a comprehensive explanation of psychological safety based on evidence from neurophysiology, psychology, and evolutionary theory (Porges, 2011), could be utilized in such a study. PVT has been used to inform the development of safety scales, such as Morton et al.’s (2022) NPSS. As trauma and resilience are two interrelated concepts that pertain to an individual’s response and coping mechanisms in the face of adversity, future research could concentrate on gaining a comprehensive comprehension of psychological safety and its relevance in the context of trauma. A study of this nature should strive to include a diverse and representative sample of individuals, including those who have and have not experienced trauma. This will enable a broader understanding of the effects of psychological stability in trauma-related settings.

## Data availability statement

The datasets presented in this article are not readily available because data was collected from minors with consent for use by REPSSI-affiliated researchers only. Requests to access the datasets should be directed to monica.bandeira@repssi.org.

## Ethics statement

The studies involving humans were approved by the University of Pretoria, Faculty of Education, Ethics Committee. The studies were conducted in accordance with the local legislation and institutional requirements. Written informed consent for participation in this study was provided by the participants’ legal guardians/next of kin.

## Author contributions

MB conducted the analyses and led a first draft of the manuscript. LE and MG assisted with conceptualizing the study focus and contribution to the knowledgebase, interpreting the study results, and collaborated in drafting and revising the manuscript. All authors read and approved the final manuscript.
